# Supplementary material for: Influence of Interactions between Nitrogen, Phosphorus Supply and Epichloё bromicola on Growth of Wild Barley (Hordeum brevisubulatum)
Source: J Fungi (Basel). 2021 Jul 29;7(8):615. doi: 10.3390/jof7080615 (PMC8397062; doi:10.3390/jof7080615)
Supplement: Supplementary file 1 [file jof-07-00615-s001.zip › Description of Figure S1.pdf]

Figure S1. Contents of Na^+ and K^+ of *Hordeum brevisubulatum* under different concentrations of N and P. (**a**) Content of Na^+, (**b**) Content of K^+. E+: *Epichloë bromicola*-infected plants, E-: *E. bromicola*-free plants. Different lowercase letters and majuscule letters on top of bars indicate significant differences ($P < 0.05$) of E- and E+ plants between the different N concentrations, respectively. An * on the top of the bars, means a significant difference at $P < 0.05$ (independent *t*-test) between E+ and E- plants under the same nutrient concentration.
