# Supplementary material for: Influence of Interactions between Nitrogen, Phosphorus Supply and Epichloё bromicola on Growth of Wild Barley (Hordeum brevisubulatum)
Source: J Fungi (Basel). 2021 Jul 29;7(8):615. doi: 10.3390/jof7080615 (PMC8397062; doi:10.3390/jof7080615)
Supplement: Supplementary file 1 [file jof-07-00615-s001.zip › Table S1.pdf]

Table S1. Three-way ANOVA for the effects of endophyte (E), nitrogen concentration (N) and phosphorus concentration (P) on germination rate (%), germination potential (%), germination index and radicle length of *Hordeum brevisubulatum*. N×P: interaction of N and P, N×E: interaction of N and *Epichloë bromicola* ; P×E: interaction of P and *E. bromicola;* N×P×E: interaction of N, P and *E. bromicola.*

**Treatments**	**dF**	**Germination rate**		**Germination potential**		**Germination index**		**Radicle length**	
		**F**	**P**	**F**	**P**	**F**	**P**	**F**	**P**
**N**	2	5.766	0.007	35.081	<0.001	47.708	<0.001	77.757	<0.001
**P**	2	1.255	0.297	0.864	0.430	0.043	0.958	0.039*	0.962
**E**	1	3.457	0.071	16.329	<0.001	20.245	<0.001	10.085	0.002
**N×P**	4	1.800	0.150	1.118	0.363	2.404	0.068	35.07	<0.001
**N×E**	2	2.313	0.113	5.771	0.007	4.084	0.025	14.281	<0.001
**P×E**	2	1.644	0.207	3.688	0.035	2.971	0.064	6.056	0.002
**N×P×E**	4	2.103	0.101	2.372	0.071	1.447	0.239	5.957	<0.001
