# Supplementary material for: Influence of Interactions between Nitrogen, Phosphorus Supply and Epichloё bromicola on Growth of Wild Barley (Hordeum brevisubulatum)
Source: J Fungi (Basel). 2021 Jul 29;7(8):615. doi: 10.3390/jof7080615 (PMC8397062; doi:10.3390/jof7080615)
Supplement: Supplementary file 1 [file jof-07-00615-s001.zip › Table S2.pdf]

Table S2. Three-way ANOVA for the effects of nitrogen concentration (N), phosphorus concentration (P) and endophyte (E) on aboveground biomass, underground biomass, plant height and number of tillers of *Hordeum brevisubulatum.* N×P: interaction of N and P, N×E: interaction of N and *Epichloë bromicola;* P×E: interaction of P and *E. bromicola;* N×P×E: interaction of N, P and *E. bromicola.*

Treatments	dF	Aboveground biomass		Underground biomass		Plant height		Number of tillers	
		F	P	F	P	F	P	F	P
**N**	2	57.684	<0.001	9.919	<0.001	173.007	<0.001	72.580	<0.001
**P**	2	66.365	<0.001	51.919	<0.001	150.507	<0.001	54.612	<0.001
**E**	1	0.298	0.587	1.124	0.293	5.670	0.021	2.621	0.107
**N×P**	4	20.093	<0.001	7.017	<0.001	22.952	<0.001	12.188	<0.001
**N×E**	2	1.585	0.212	0.769	0.467	2.007	0.144	1.018	0.363
**P×E**	2	0.671	0.515	2.095	0.130	1.017	0.368	2.031	0.133
**N×P×E**	4	0.423	0.792	6.558	<0.001	2.089	0.095	0.451	0.772
