# Supplementary material for: Influence of Interactions between Nitrogen, Phosphorus Supply and Epichloё bromicola on Growth of Wild Barley (Hordeum brevisubulatum)
Source: J Fungi (Basel). 2021 Jul 29;7(8):615. doi: 10.3390/jof7080615 (PMC8397062; doi:10.3390/jof7080615)
Supplement: Supplementary file 1 [file jof-07-00615-s001.zip › Table S3.pdf]

Table S3. Three-way ANOVA for the effects of nitrogen concentration (N), phosphorus concentration (P) and endophyte (E) on a chlorophyll content and root metabolic activity of *Hordeum brevisubulatum.* N×P: interaction of N and P, N×E: interaction of N and *Epichloë bromicola;* P×E: interaction of P and *E. bromicola;* N×P×E: interaction of N, P and *E. bromicola.*

**Treatments**	**dF**	**Chlorophyll content**		**Root metabolic activity**	
		**F**	**P**	**F**	**P**
**N**	2	34.256	<0.001	6.579	0.004
**P**	2	5.355	0.009	46.398	<0.001
**E**	1	32.346	<0.001	18.774	<0.001
**N×P**	4	7.314	<0.001	25.352	<0.001
**N×E**	2	2.042	0.145	2.319	0.113
**P×E**	2	2.49	0.097	14.638	<0.001
**N×P×E**	4	2.628	0.051	3.859	0.010
