# Supplementary material for: Influence of Interactions between Nitrogen, Phosphorus Supply and Epichloё bromicola on Growth of Wild Barley (Hordeum brevisubulatum)
Source: J Fungi (Basel). 2021 Jul 29;7(8):615. doi: 10.3390/jof7080615 (PMC8397062; doi:10.3390/jof7080615)
Supplement: Supplementary file 1 [file jof-07-00615-s001.zip › Table S4.pdf]

Table S4. Three-way ANOVA for the effects of nitrogen concentration (N), phosphorus concentration (P) and endophyte (E) on Na^{+} and K^{+} contents in leaves and roots of *Hordeum brevisubulatum.* N×P: interaction of N and P, N×E: interaction of N and *Epichloë bromicola ;* P×E: interaction of P and *E. bromicola;* N×P×E: interaction of N, P and *E. bromicola.*

	Treatments	dF	Na^{+} content		K^{+} content	
			F	P	F	P
Aboveground	**N**	2	2.327	0.112	94.271	<0.001
	**P**	2	49.234	<0.001	41.616	<0.001
	**E**	1	1.765	0.192	0.028	0.868
	**N×P**	4	3.976	0.009	13.793	<0.001
	**N×E**	2	5.575	0.008	23.738	<0.001
	**P×E**	2	6.592	0.004	18.902	<0.001
	**N×P×E**	4	3.049	0.029	2.089	0.105
Underground	**N**	2	123.497	<0.001	61.109	<0.001
	**P**	2	111.835	<0.001	136.893	<0.001
	**E**	1	5.373	0.027*	14.872	<0.001
	**N×P**	4	39.533	<0.001	15.442	<0.001
	**N×E**	2	17.62	<0.001	4.248	0.023
	**P×E**	2	10.926	<0.001	2.925	0.068
	**N×P×E**	4	6.854	<0.001	4.234	0.007
