# Supplementary figures and images for: Influence of Interactions between Nitrogen, Phosphorus Supply and Epichloё bromicola on Growth of Wild Barley (Hordeum brevisubulatum)
Source: J Fungi (Basel). 2021 Jul 29;7(8):615. doi: 10.3390/jof7080615 (PMC8397062; doi:10.3390/jof7080615)

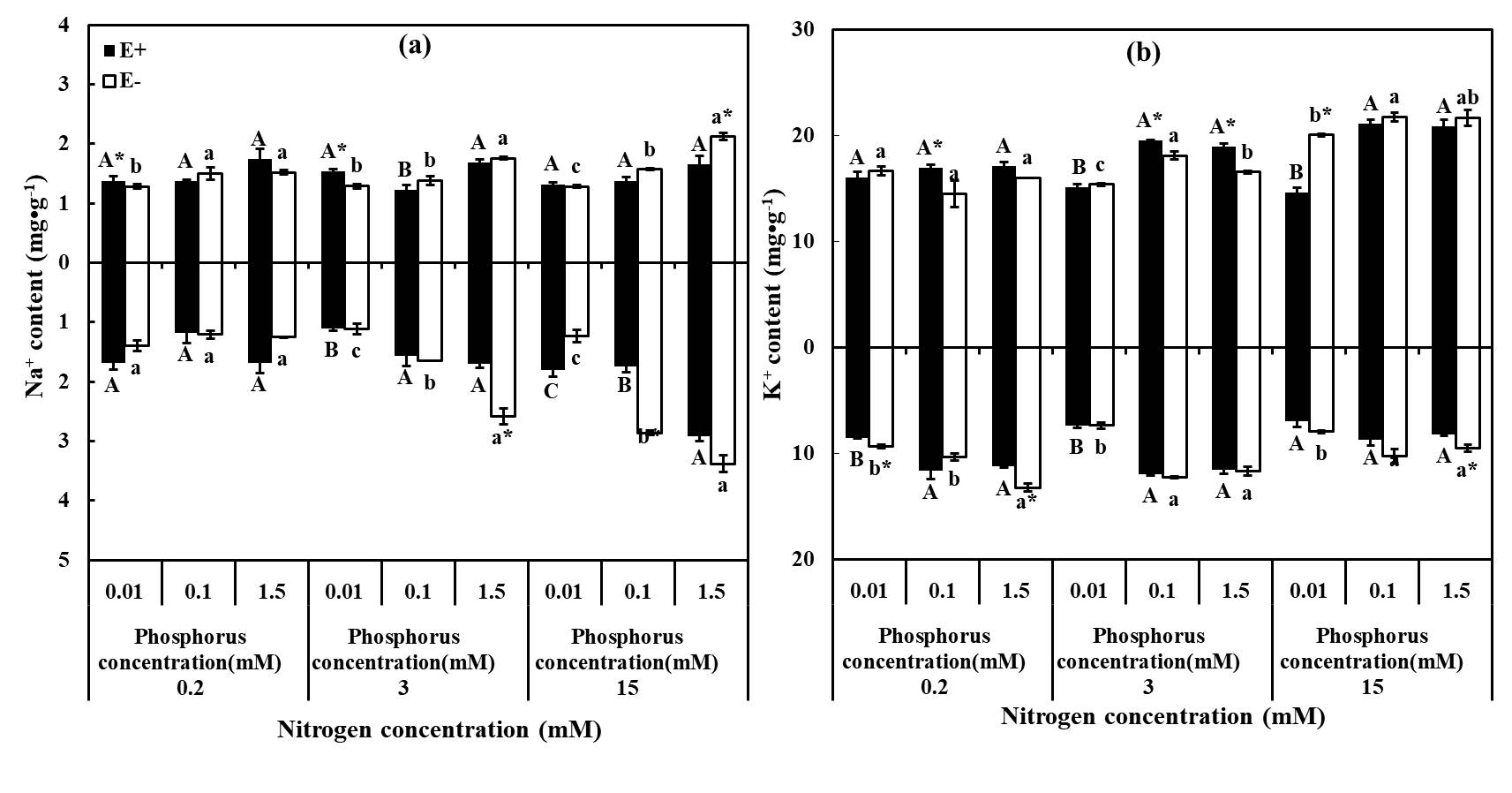

Supplement: Supplementary file 1 [file jof-07-00615-s001.zip › Figure S1.jpg]
